# Joint association of overweight/obesity, high electronic screen time, and low physical activity time with early pubertal development in girls: a case–control study

**DOI:** 10.1038/s41598-024-60345-7

**Published:** 2024-05-08

**Authors:** Weiqin Li, Lingyan Feng, Panpan Song, Leishen Wang, Shuang Zhang, Wei Li, Dandan Zhu, Yuexin Du, Junhong Leng

**Affiliations:** 1 Tianjin Women and Children’s Health Center, 96 Guizhou Road, Heping District, Tianjin, 300070 China; 2 School of Public Health and Health Sciences, Tianjin University of Traditional Chinese Medicine, 10 Poyang Lake Road, Jinghai District, Tianjin, 301617 China

**Keywords:** Overweight, Electronic screen time, Physical activity, Early pubertal development, Children, Health care, Risk factors

## Abstract

To examine the joint association of electronic screen time (EST), moderate-to-vigorous physical activity time (MVPA) and overweight/obesity with early pubertal development (EPD) in girls. A case–control study of 177 EPD girls and 354 girls with normal pubertal development was conducted between October 2019 and August 2022. Overweight/obesity was defined as body mass index ≥ 85th percentiles for age and sex. We found a non-significant increase of EPD risk among girls with high EST alone [OR: 2.75 (0.65–11.58)] or low MVPA alone [OR: 2.54 (0.74–8.69)], but a significant increase of EPD risk among girls with overweight/obesity alone [OR: 4.91 (1.01–23.92)], compared to girls without any of the three risk factors (low MVPA, high EST and overweight/obesity). Girls with any two of the three risk factors faced increased risk of EPD, and girls with all three risk factors faced the highest risk of EPD [OR and 95% CI: 26.10 (6.40–106.45)]. Being overweight/obesity might be more important than having low MVPA or high EST as a correlate of EPD compared to girls without any of the three risk factors, but the co-presence of low MVPA, high EST and overweight/obesity would largely increase the risk of EPD in girls.

## Introduction

Early pubertal development (EPD) is a common endocrine disease among children, characterized by early secondary sexual development and early menarche. The prevalence of EPD is increasing worldwide, which is more frequent in girls. Observational data from the US showed that at age 7, 10% of white girls and 23% of black girls have started puberty^1^. According to a school-based survey in China, 11.47% of girls and 3.26% of boys had signs of pubertal development before age 8 and 9^2^, with a markedly higher occurrence in girls than in boys. During the pandemic of COVID-19, a few recent studies reported that the incidence of precocious puberty increased and puberty accelerated^3–6^. The experience of EPD can lead to accelerated skeletal maturation, advanced bone age, and early epiphyseal closure, all of which can impact final adult height. In addition, it may result in psychological issues or abnormal social behavior^7^. The long-term health consequences associated with early menarche include increased risk of obesity, type 2 diabetes, estrogen-dependent cancers, and cardiovascular events^8^. Therefore, EPD has raised global public health concerns, and strategies to prevent EPD are highlighted worldwide.

Childhood obesity is one of the global public health challenges^9^. The global age-standardized prevalence of obesity increased from 0.7% in 1975 to 5.6% in 2016 in girls, and from 0.9% in 1975 to 7.8% in 2016 in boys^10^. According to the World Health Organization, more than 390 million children and adolescents aged 5 to 19 were overweight in 2022, including 160 million who were living with obesity^11^. Previous studies have observed that early-onset puberty is often associated with excess body weight, especially in girls^12–14^. Lifestyle habits including physical activity and electronic screen time (EST) are also potential influencing factors of EPD^15–17^, but the evidence are still insufficient. A study presented at the 60^th^ Annual European Society for Pediatric Endocrinology Meeting in Rome suggested that the increase of precocious puberty during the pandemic of COVID-19 might be resulted from unhealthy lifestyles including long time spent on smart devices^18^. Until now, there have been no studies assessing the joint association of high EST, low physical activity time and overweight/obesity with EPD in girls.

## Materials and methods

### Study design and participants

This is a case–control study with 177 EPD girls and 354 girls with normal pubertal development conducted at Tianjin Women and Children's Health Center from October 2019 to August 2022. This study has been approved by the Ethics Committee of Tianjin Women and Children Health Center (approval number: ky-20190119), and all guardians of the study participants signed informed consent. All methods were performed in accordance with the relevant guidelines and regulations.

Girls who were newly diagnosed to have EPD (7.72 ± 1.0 years old) at Tianjin Women and Children's Health Center were invited to participate the study, and then they and their guardians were interviewed. Tianjin Women and Children's Health Center is a city-level public hospital, and provides health care services to women and children in Tianjin. The inclusion criteria of case group were as follows: (1) Girls. (2) Confirm the Chinese diagnostic criteria^19^ for EPD in girls, which involves secondary sexual characteristics development before 8 years old or menstruation before 10 years old. All girls were physically examined in a private room by qualified female pediatricians, and then were given a B-ultrasound test of breast, uterus and ovary using an ultrasound Philips EPIQ7. The Tanner Staging was applied to evaluate children’s secondary sexual characteristics^20,21^. (3) Children and their guardians agreed to participate and signed informed consent. Exclusion criteria include: (1) Secondary central precocities, such as central nervous system occupying, infection, trauma, postoperative, radiotherapy or chemotherapy, congenital dysplasia, etc. (2) Other primary diseases that may lead to EPD, such as congenital adrenal hyperplasia, McCune-Albright syndrome, congenital hypothyroidism, granulomatous disease, malignant tumor, and cerebral palsy. (3) A history of hormone drug use.

A total of 354 girls who exhibited normal pubertal development of the same age range (7.60 ± 1.0) were recruited as the control group, based on the routine physical examination of Tianjin kindergartens/primary school students. To achieve a satisfactory power of test, the number of participants in the control group was twice of the case group. The inclusion criteria for the control group were as follows: (1) With normal pubertal development and without secondary sexual characteristics development before 8 years old. All girls were physically examined in a private room by qualified female pediatricians, and the Tanner Staging was applied to evaluate children’s secondary sexual characteristics. (2) Children and their guardians agreed to participate and signed informed consent. Exclusion criteria include: (1) Primary diseases that may lead to EPD, such as congenital adrenal hyperplasia, McCune-Albright syndrome, congenital hypothyroidism, granulomatous disease, malignant tumor, and cerebral palsy. (2) A history of hormone drug use.

### Data collection methods

We obtained information about the participants by using a questionnaire. The investigator filled out the questionnaires during a face-to-face interview with the children and their guardians. The questionnaire was conducted by the researchers after reviewing the current evidence and revised by several pediatricians based on their expertise (a copy of the questionnaire was presented in the Supplementary Material 1). The questionnaire contained five sections including: (1) General information (age, date of birth, and ethnicity); (2) Parental information (height and weight of parents, mother’s age of first menarche, and father’s age of first spermatogenesis); (3) Diet information in 1 month prior to the survey and the average per week was calculated, by asking “How many eggs did you eat, including all kinds of cooking like poaching, frying, and being included in other foods ?”, “How often did you eat snacks, including chips, cookies, candy, cake, chocolate, ice cream and other desserts ?”; (4) Behavior information in 1 month prior to the survey, and the average time per week was calculated, including: electronic screen watching time, MVPA time, and time spent on the roadside (including parking, walking, or biking on the roadside); the proportion of plastic bottled water in total drinking water was also surveyed. Moderate physical activity is defined as levels 12–14 of 20 on the RPE scale^22^, and the intensity is 3.0 to 5.9 metabolic equivalent (MET), such as jogging, skating, cycling at normal speed, etc. Vigorous physical activity is defined as levels 15 or above of 20 on the RPE scale, and the intensity is ≥ 6 MET, such as carrying heavy objects, running fast, cycling fast, etc.; (5) Sleep habits in 1 month prior to the survey, and the average time per day was calculated, including: wakeup time in the morning, bedtime at night, average time of night sleeping, and average time of day sleeping. The total sleeping time was calculated by night sleeping time plus day sleeping time.

Children’s height, weight, and waist circumference were measured according to standardized procedures. Body weight was measured to the nearest 0.01 kg by using a digital scale (TCS-60, Tianjin Weighing Apparatus Co., China). Standing height was measured to the nearest 0.1 cm using a stadiometer (SZG-180, Shanghai Zhengdahengqi, China). Children’s waist circumference was measured midway between the lower rib margin and the iliac crest, and the measurement was rounded to the nearest 0.1 cm. Children’s body fat percentage was measured using a body composition analyzer (Inbody J-20, South Korea). Body mass index (BMI) was obtained by dividing weight in kilograms by the square of height in meters. Children’s Z scores of BMI for age were calculated according to the WHO age- and gender-specific growth reference (0 ~ 60 months^23^ and 5 ~ 19 years old^24^. Childhood overweight/obesity was defined as BMI ≥ 85th percentiles (Z score of BMI for age ≥ 1.035) which included both overweight and obesity to improve the statistical power of test.

### Statistical analyses

The criterion of statistical significance was < 0.05 (for two-sided tests). All statistical analyses used IBM SPSS Statistics 24.0 (IBM SPSS, Chicago, IL). The general characteristics between groups were compared by using student’s *t*-test for continuous variables if their normal distribution was not rejected, and Chi-square test for categorical variables.

Binary logistic regression models were used to obtain odds ratios (ORs) and their 95% confidence intervals (CIs) of overweight/obesity, EST and MVPA as categorical variables (normal weight [reference], and overweight/obesity; low EST as EST ≤ 7 h/week [reference], and high EST as EST > 7 h/week; low MVPA as MVPA ≤ 7 h/week, and high MVPA as MVPA > 7 h/week [reference]) with the risks of EPD. We included two models in the logistic analyses: Model 1, univariate analyses. Model 2, multivariate analyses adjusted for mother's age of menarche, father's age of first spermatogenesis, age, time on the roadside, proportion of plastic bottled water in total drinking water, night sleep time, and day sleep time.

The period of data collection included both of the periods before (between October 2019 and December 2019) and during (between January 2020 and August 2022) the COVID-19 Pandemic, and this confinement period might be a strong influencing factor. However, only 13 EPD girls and 62 controlled girls were recruited before the COVID-19 Pandemic, so we did a sensitivity analysis by excluding participants who were recruited before the COVID-19 Pandemic.

### Ethical approval

The studies involving human participants were reviewed and approved by the Ethics Committee of Tianjin Women and Children’s Health Center (approval number: ky-20190119). Written informed consent to participate in this study was provided by the participants’ legal guardians.

## Results

### General characteristics

In all participants, the age of girls in the case group and the control group was 7.72 ± 1.0 years and 7.60 ± 1.0 years, respectively, with no statistical difference between the two groups (*t* = − 1.300, *P* = 0.194). According to the general information shown in Table 1, there were significant differences of mother's age of menarche, father's age of first spermatogenesis, height, weight, BMI, waist circumference, EST, MVPA, time on the roadside, proportion of plastic bottled water in total drinking water, bedtime at night, night sleeping time, day sleeping time, and total sleeping time (all *P* values < 0.05). In the sensitivity analyses after excluding participants who were recruited before the COVID-19 Pandemic, girls in the case control group was older than those in the control group, and other comparison between the two groups almost did not change.

**Table 1:** General information for the case and control groups.

	All participants			Participants recruited during the COVID-2019 pandemic^#^		
	Case group (n = 177)	Control group (n = 354)	*P-*value	Case group (n = 164)	Control group (n = 292)	*P-*value
Age (years)	7.72 ± 1.0	7.60 ± 1.0	0.194	7.75 ± 1.0	7.42 ± 0.9	0.001
**Information of parents**						
Mother's age (years)	36.41 ± 3.7	36.48 ± 4.4	0.840	36.39 ± 3.8	36.47 ± 4.8	0.843
Mother's BMI (kg/m2)	23.72 ± 3.6	23.47 ± 3.9	0.471	23.70 ± 3.7	23.42 ± 3.8	0.436
Mother's age of menarche (years)	12.31 ± 1.3	12.82 ± 0.7	< 0.001	12.35 ± 1.3	12.83 ± 0.7	< 0.001
Father's age (years)	37.57 ± 4.4	37.57 ± 4.8	0.995	37.49 ± 4.4	37.50 ± 5.2	0.980
Father's BMI (kg/m^2^)	25.95 ± 3.6	26.23 ± 4.6	0.485	25.98 ± 3.5	26.48 ± 4.7	0.193
Father's age of first spermatogenesis (years)	14.06 ± 1.1	14.20 ± 1.0	< 0.001	14.03 ± 1.0	14.20 ± 1.1	< 0.001
**Physical examination**						
Height (cm)	131.71 ± 10.0	127.53 ± 8.2	< 0.001	131.60 ± 9.7	126.38 ± 7.5	< 0.001
Weight (kg)	30.84 ± 9.0	27.23 ± 7.1	< 0.001	30.78 ± 8.9	26.60 ± 6.5	< 0.001
BMI (kg/m^2^)	17.47 ± 3.1	16.54 ± 2.8	0.001	17.48 ± 3.1	16.48 ± 2.7	< 0.001
Z scores for BMI	0.67 ± 1.3	0.26 ± 1.3	< 0.001	0.67 ± 1.3	0.27 ± 1.2	0.001
Waist circumference (cm)	62.28 ± 7.2	56.76 ± 7.1	< 0.001	62.22 ± 7.0	56.12 ± 6.8	< 0.001
Body fat percent (%)*	22.54 ± 6.2	22.47 ± 4.7	0.899	22.70 ± 6.2	22.22 ± 3.4	0.373
**Diet information**						
Eggs ≥ 250 g/week	115 (65.0)	217 (61.3)	0.410	94 (69.6)	172 (60.6)	0.072
Sweet snacks ≥ 3 times/week	112 (63.3)	201 (56.8)	0.151	99 (73.3)	232 (81.7)	0.051
**Behavior information**						
EST > 7 h/week	98 (55.4)	111 (31.4)	< 0.001	95 (57.9)	103 (35.3)	< 0.001
MVPA ≤ 7 h/week	153 (86.4)	232 (65.5)	< 0.001	136 (82.9)	183 (62.7)	< 0.001
Time on the roadside (min/day)	13.61 ± 12.6	11.23 ± 7.5	0.021	13.68 ± 13.0	10.86 ± 7.5	0.011
Proportion of plastic bottled water in total drinking water (%)	28.74 ± 38.8	10.13 ± 22.4	< 0.001	28.18 ± 38.4	9.86 ± 23.4	< 0.001
**Sleeping information**						
Wakeup time in the morning (AM)	7.04 ± 0.6	7.03 ± 0.4	0.831	7.05 ± 0.6	7.01 ± 0.4	0.532
Bedtime at night (PM)	9.75 ± 0.6	9.61 ± 0.5	0.004	9.75 ± 0.6	9.60 ± 0.6	0.007
Night sleeping time (hours/day)	9.29 ± 0.7	9.42 ± 0.6	0.027	9.30 ± 0.8	9.41 ± 0.6	0.083
Day sleeping time (min/day)	26.19 ± 34.6	51.83 ± 21.6	< 0.001	25.68 ± 34.9	53.90 ± 21.6	< 0.001
Total sleeping time (hours/day)	9.73 ± 1.0	10.29 ± 0.7	< 0.001	9.72 ± 1.0	10.31 ± 0.7	< 0.001

The *t*-test was used to compare the continuous variables and the results were presented in mean ± SD. The χ^2^ test was used to compare the categorical variables and the results were presented as frequency and percentage (%).

*EST* electronic screen time, *MVPA* moderate-to-vigorous physical activity.

*Data of body fat percent was missing for 19.8% (35/177) in the case group and 57.0% (200/351) in the control group.

^#^Sensitivity analysis, by excluding participants who were recruited before the COVID-19 Pandemic.

### Independent association of overweight/obesity, low MVPA and high EST with EPD

EPD was independently associated with overweight/obesity, low MVPA and high EST in both univariate and multivariate analyses (Table 2), and the multivariable-adjusted ORs and 95% CIs were 2.41 (1.43–4.06), 3.12 (1.72–5.65), and 4.01 (2.44–6.57) respectively as compared to their reference counterparts. In the sensitivity analyses after excluding participants who were recruited before the COVID-19 Pandemic, results almost did not change.

**Table 2:** Odds ratios and 95% confidence intervals of electronic screen time and physical activity for precocious puberty.

	No. of cases	No. of control	Odds ratios (95% confidence intervals)	
			Univariate analyses	Multivariate analyses*
**All participants**	177	354		
Overweight				
Normal weight (ref)	107	267	1.00	1.00
Overweight	70	87	2.01 (1.36–2.95)	2.41 (1.43–4.06)
MVPA				
≤ 7 h/week	149	232	2.80 (1.77–4.43)	3.12 (1.72–5.65)
> 7 h/week (ref)	28	122	1.00	1.00
EST				
≤ 7 h/week (ref)	76	243	1.00	1.00
> 7 h/week	101	111	2.91 (2.00–4.22)	4.01 (2.44–6.57)
**Participants recruited during the COVID-2019 Pandemic**^#^	164	292		
Overweight				
Normal weight (ref)	100	222	1.00	1.00
Overweight	64	70	2.01 (1.33–3.04)	2.34 (1.32–4.16)
MVPA				
≤ 7 h/week	136	183	2.91 (1.82–4.67)	3.37 (1.79–6.35)
> 7 h/week (ref)	28	109	1.00	1.00
EST				
≤ 7 h/week (ref)	69	189	1.00	1.00
> 7 h/week	95	103	2.49 (1.68–3.68)	2.90 (1.69–4.96)

*EST* electronic screen time, *MVPA* moderate-to-vigorous physical activity.

*Adjusted for mother's age of menarche, father's age of first spermatogenesis, age, time on the roadside, proportion of plastic bottled water in total drinking water, night sleep time, and day sleep time.

^#^Sensitivity analysis, by excluding participants who were recruited before the COVID-19 Pandemic.

### Joint association of overweight/obesity, high EST and low MVPA with EPD

**Figure 1:**
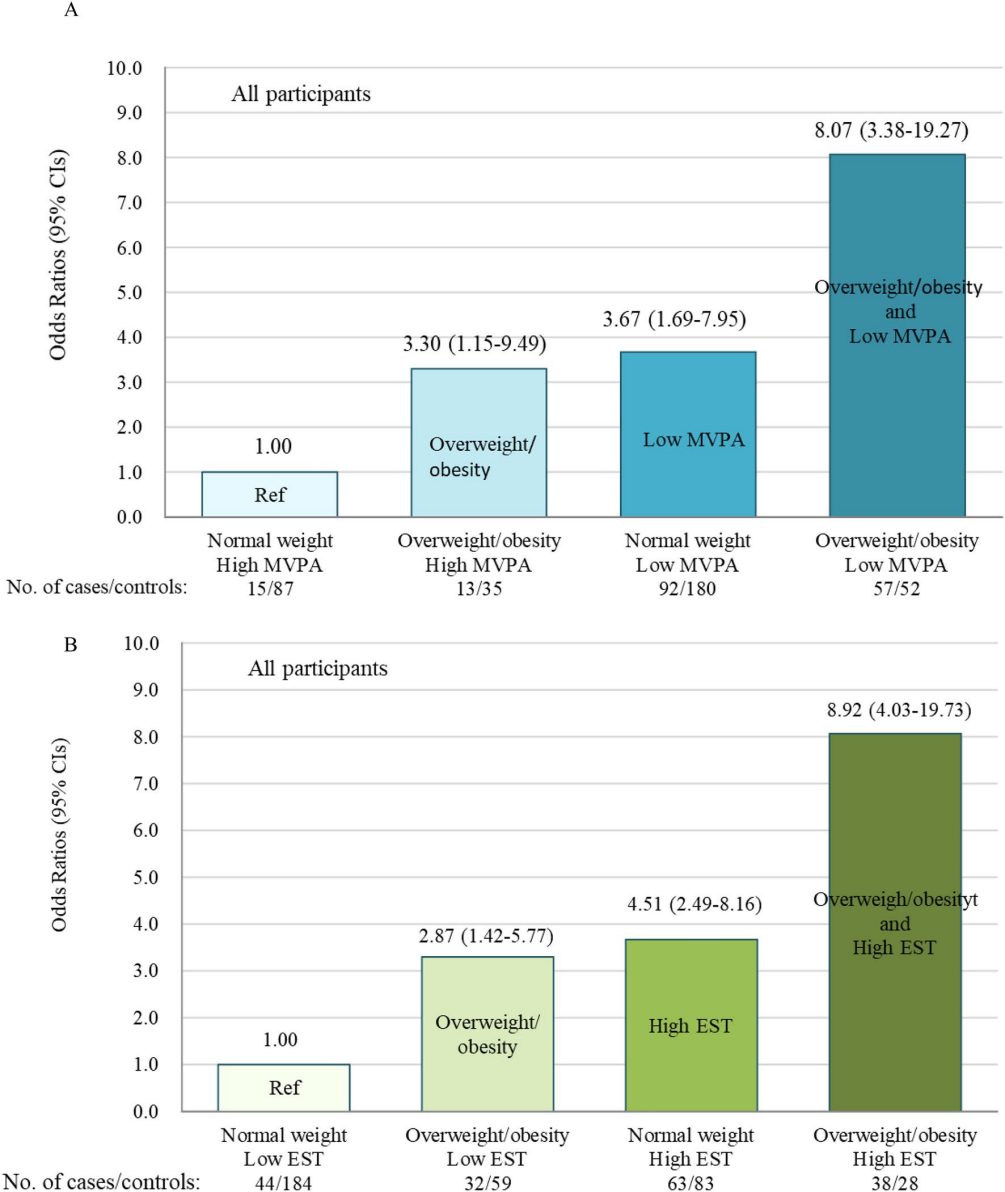
Relative contributions from exposures to overweight/obesity, low *MVPA* and both (**A**), and overweight/obesity, high EST and both (**B**) to the risk of EPD in all participants. *MVPA* moderate-to-vigorous physical activity, *EST* electronic screen time.

The relative contributions of exposures to overweight/obesity and low MVPA and both for the risk of EPD were showed in Fig. 1A. Using girls with normal weight and high MVPA as the referent group, the OR of EPD increased from 1.00 to 3.30 (1.15–9.49) for the isolated presence of overweight/obesity, and to 3.67 (1.69–7.95) for the isolated presence of low MVPA, and to 8.07 (3.38–19.27) for the co-presence of overweight/obesity and low MVPA. The relative contributions of exposures to overweight/obesity and high EST and both for the risk of EPD were shown in Fig. 1B. Compared to girls with normal weight and low EST, the OR of EPD increased from 1.00 to 2.87 (1.42–5.77) for the isolated presence of overweight/obesity, and to 4.51 (2.49–8.16) for the isolated presence of high EST, and to 8.92 (4.03–19.73) for the co-presence of overweight/obesity and high EST. In the sensitivity analyses after excluding participants who were recruited before the COVID-19 Pandemic, results almost did not change (Supplementary Material 2).

**Figure 2:**
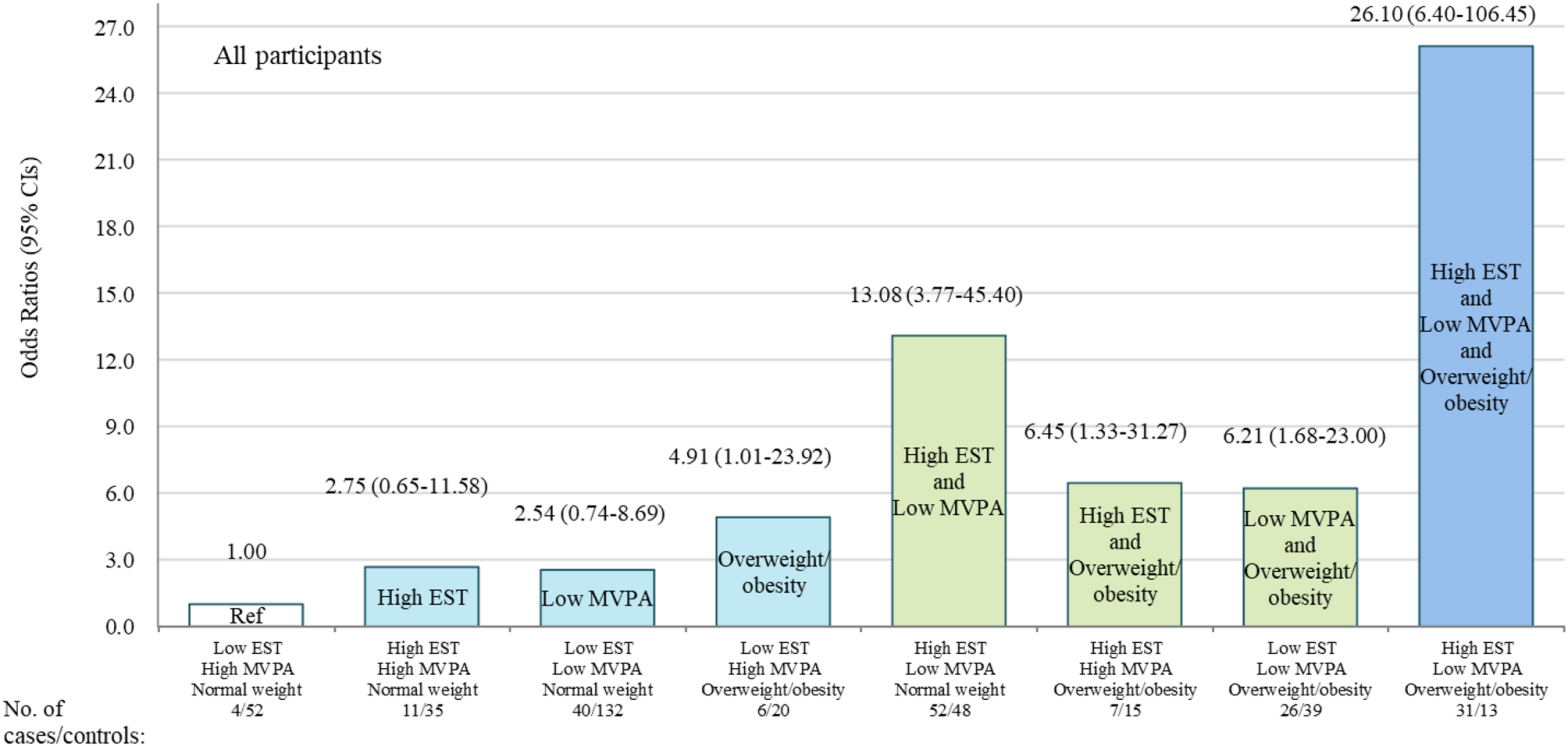
Joint effects overweight/obesity, low *MVPA* and high EST on the risk of *EPD* in all participants.

**Figure 3:**
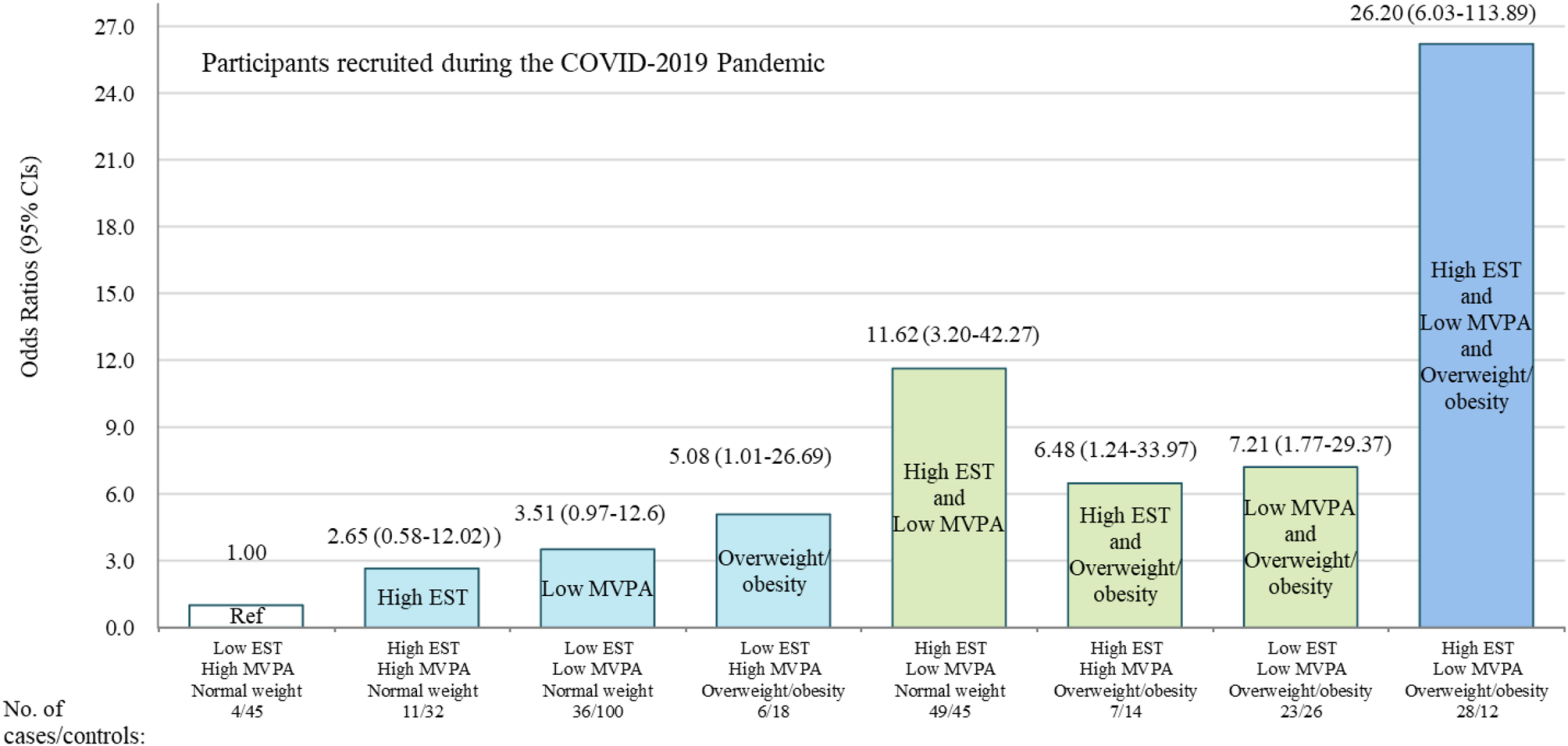
Joint effects overweight/obesity, low *MVPA* and high *EST* on the risk of *EPD* in Participants recruited during the COVID-2019 Pandemic.

To examine the joint association of overweight/obesity, high EST and low MVPA with EPD, girls were divided into eight groups, and those without any of the three risk factors (low MVPA, high EST and overweight/obesity) were designated as the reference group (Fig. 2). We found a non-significant increase of EPD risk among girls with high EST alone [OR: 2.75 (0.65–11.58)] or low MVPA alone [OR: 2.54 (0.74–8.69)], but a significant increase of EPD risk among girls with overweight/obesity alone [OR: 4.91 (1.01–23.92)], compared to girls without any of the three risk factors (low MVPA, high EST and overweight/obesity). Girls with any two of the three risk factors faced increased risk of EPD, with ORs and 95% CIs of 13.08 (3.77–45.40) for the co-presence of high EST and low MVPA, 6.45 (1.33–31.27) for the co-presence of high EST and overweight/obesity, and 6.21 (1.68–23.00) for the co-presence of low MVPA and overweight/obesity. Girls with all of the three risk factors faced the highest risk of EPD [OR and 95% CI: 26.10 (6.40–106.45)]. In the sensitivity analyses after excluding participants who were recruited before the COVID-19 Pandemic, results almost did not change (Fig. 3).

## Discussion

In this case–control study, overweight/obesity, low MVPA and high EST were all risk factors for EPD in girls. We found a non-significant increase of EPD risk among girls with high EST alone or low MVPA alone, but a significant increase of EPD risk among girls with overweight/obesity alone, compared to girls without any of the three risk factors. Being overweight/obesity might be more important than having low MVPA or high EST as a correlate of EPD compared to girls without any of the three risk factors, but the co-presence of low MVPA, high EST and overweight/obesity would largely increase the risk of EPD in girls.

Childhood obesity and EPD are both major public health problems worldwide, and the relationship between childhood obesity and early onset of puberty has drawn more and more attention. The present study confirmed the results of previous studies that increased adiposity is associated with earlier onset of puberty in girls^25^. As this association is more complex in boys^25^ and the prevalence of EPD is higher in girls than in boys, we only focused on girls in the present study. The mechanisms underlying this association of obesity with EPD remain elusive. A recent review summarized the recent progress regarding how childhood obesity impacts on hypothalamic-pituitary–gonadal (HPG) axis and pubertal onset^12^. Simply, the increase of fat can promote the secretion of leptin, ghrelin, insulin and other hormones, all of which can stimulate the hypothalamus and promote the development of gonads, leading to the onset of puberty**.**

After the outbreak of the novel COVID-19, China and other countries globally enacted lockdown policies as an epidemiological containment strategy to reduce the spread of COVID-19 infection. During the lockdown, unhealthy lifestyles and behaviors were observed including over-eating or skipping meals, sleep problems, decreased physical activity, increased use of screening devices, changes in body weight, smoking behavior, and alcohol consumption^26,27^. At the same time during the pandemic and lockdown for COVID-19, the incidence of EPD increased and puberty accelerated in female^28,29^. The period of data collection in our study included both of the periods before and during the COVID-19 Pandemic. As a case–control study, it aimed not to compare the exact rates of EPD and unhealthy behaviors between the periods before and during the COVID-19 Pandemic, but to examine the association of EST, MVPA and overweight/obesity with EPD in girls. This confinement period might be a strong influencing factor, so we did a sensitivity analysis by excluding participants who were recruited before the COVID-19 Pandemic. The results of sensitivity analyses showed similar effects of EST, MVPA and overweight/obesity on EPD with those in total participants. Thus, we suggested that high EST, low MVPA and overweight/obesity would increase the EPD risk regardless of the COVID-19 Pandemic. This finding was consistent with the review presented at the 60^th^ Annual European Society for Pediatric Endocrinology Meeting in Rome, which suggested that the increase of EPD during the pandemic of COVID-19 might not have anything to do with the infection at all, and time spent during lockdowns scrolling through smart devices for hours on end could be to blame^18^.

The present study found that the risk of EPD increased three times in girls who spent more than 7 h per week on electronic screen, and it supported the above view^18^. The mechanisms of light exposure correlating with early signs of maturity have not been confirmed among human beings, but they have been explained by animal studies, including sleep disruption and melatonin theory^15^. Sleep disruption could result in lack of sleep which is correlated with weight gain, and weight gain is correlated with early puberty^15^. Light exposure could reduce the secretion of melatonin, and then advance the secretion of pituitary gonadotropin which was normally suppressed by melatonin, leading to EPD^15^.

In this study, we also found that the risk of EPD increased more than two times in girls who did not exercise enough (MVPA ≤ 7 h/week). The main mechanism is that physical exercise specifically inhibits the pulsating release of hypothalamic gonadotropin-releasing hormone (GnRH), restricts the pituitary secretion of luteinizing hormone and, to a lesser extent, follicle-stimulating hormone, thereby limiting ovarian stimulation^16^. At the same time, physical activity may delay puberty by reducing the synthesis of sex hormones by reducing obesity, or by decreasing insulin levels, increasing sex hormone-binding globulin levels, and decreasing estradiol bioavailability^17^.

There are no studies assessing the joint association of overweight/obesity, EST and physical activity with EPD previously. Our findings regarding the joint effect of high EST, low MVPA and overweight/obesity on EPD in girls is biologically plausible. Observational studies have concluded that behaviors and lifestyles including EST and physical activity are associated with obesity. Gordon-Larsen et al. reported that obesity risk was proportional to TV-watching hours and inversely proportional to significant physical activity among adolescents^30^. Therefore, besides the potential mechanisms of light and physical activity mentioned above, both high EST and low MVPA could increase the risk of EPD by influencing obesity.

This study assessed the joint association of overweight/obesity, EST and physical activity with EPD in girls for the first time. However, it has some limitations. Firstly, most of the information including EST and MVPA were not obtained by a direct measure, but through interview questionnaires. This kind of data collection could lead to bias. Secondly, this study could not distinguish between central or peripheral EPD, as the sex hormone stimulation test was not carried out in all girls in the case group. And also not all participants underwent the bone age X-ray examination. Finally, the study participants were collected from one medical institution which might introduce selection bias. Thus, our findings may not be generalized to all populations of Chinese children. The findings of this study need to be further confirmed by future studies.

## Conclusion

In this case–control study, we found that overweight/obesity, low MVPA and high EST were all risk factors for EPD in girls. Being overweight/obesity might be more important than having low MVPA or high EST as a correlate of EPD in girls, as the isolated presence of low MVPA or high EST would not significantly increase the risk of EPD whereas the isolated presence of overweight/obesity would significantly increase the risk of EPD in girls. However, the co-presence of any two of the three risk factors would largely increase the risk of EPD in girls. The possible causes and mechanisms are still unclear, and researches are needed in the future. We recommend girls do not spend their leisure time watching TV or on other electronic screens, avoid a sedentary lifestyle which is characterized by both low MVPA and high EST, but instead spend more time exercising, for reasons related to obesity and also potential early puberty, even in girls of normal weight.

### Supplementary Information

Supplementary Information 1.

Supplementary Information 2.

## Data Availability

Data is provided within the manuscript or supplementary information files. The datasets are available from the corresponding author on reasonable request.
