# Supplementary material for: Joint association of overweight/obesity, high electronic screen time, and low physical activity time with early pubertal development in girls: a case–control study
Source: Sci Rep. 2024 May 8;14:10541. doi: 10.1038/s41598-024-60345-7 (PMC11078933; doi:10.1038/s41598-024-60345-7)
Supplement: Supplementary file 1 — Supplementary Information 1. [file 41598_2024_60345_MOESM1_ESM.pdf]

**Supplementary material 1. A copy of the questionnaire**

Number of participants:__________

| | |
|---|---|
| A. General information | |
| ● | Name: __________ |
| ● | Date of birth: ______________ |
| ● | Ethnicity: ______________ |
| B. Diet information in 1 month prior to the survey | |
| ➢ | How many eggs did you eat, including all kinds of cooking like poaching, frying, and being included in other foods? (the average number of eggs per week was estimated)<br>① ≥ 250 g/week ② < 250 g/week |
| ➢ | How often did you eat snacks, including chips, cookies, candy, cake, chocolate, ice cream and other desserts? (the average times of snacks per week were estimated)<br>① ≥ 3 times/week ② < 3 times/week |
| ➢ | Other special food?<br>________________________________________________ |
| C. Behavior information in 1 month prior to the survey | |
| ● | Electronic screen watching time (the average time was estimated):<br>① > 7 hours/week ② ≤ 7 hours/week |
| ● | Moderate-to-vigorous physical activity time (the average time was estimated):<br>① > 7 hours/week ② ≤ 7 hours/week<br>Note: Moderate physical activity is defined as levels 12-14 of 20 on the RPE scale, and the intensity is 3.0 to 5.9 metabolic equivalent (MET), such as jogging, skating, cycling at normal speed, etc.<br>Vigorous physical activity is defined as levels 15 or above of 20 on the RPE scale, and the intensity is ≥ 6 MET, such as carrying heavy objects, running fast, cycling fast, etc. |
| ● | Time spent on the roadside (including parking, walking, or biking on the roadside)<br>_______min/day (the average time was estimated) |
| ● | The proportion of plastic bottled water in total drinking<br>_______% |
| D. Sleep habits in 1 month prior to the survey | |
| ➢ | Wakeup time in the morning: _______AM |
| ➢ | Bedtime at night: _______PM |
| ➢ | Average time of night sleeping: _______hours/day |

| |
|---|
| ➢ Average time of day sleeping: _______min/day |
| ➢ Total sleeping time: _______hours/day (night sleeping time plus day sleeping time) |
| E1. Maternal information |
| ● Height: _________ cm |
| ● Date of birth: __________ |
| ● Weight: ____________ kg |
| ● Age of first menarche: __________ |
| E2. Paternal information |
| ➢ Height: __________ cm |
| ➢ Date of birth: __________ |
| ➢ Weight: ______________ kg |
| ➢ Age of first spermatogenesis: __________ |

Guardian Signature: ___________

Tel: ______________

Date: _________________
