# Supplementary material for: Joint association of overweight/obesity, high electronic screen time, and low physical activity time with early pubertal development in girls: a case–control study
Source: Sci Rep. 2024 May 8;14:10541. doi: 10.1038/s41598-024-60345-7 (PMC11078933; doi:10.1038/s41598-024-60345-7)

Supplementary material 2. Relative contributions from exposures to overweight/obesity, low MVPA and both (A), and overweight/obesity, high EST and both (B) to the risk of EPD in Participants recruited during the COVID-2019 Pandemic.

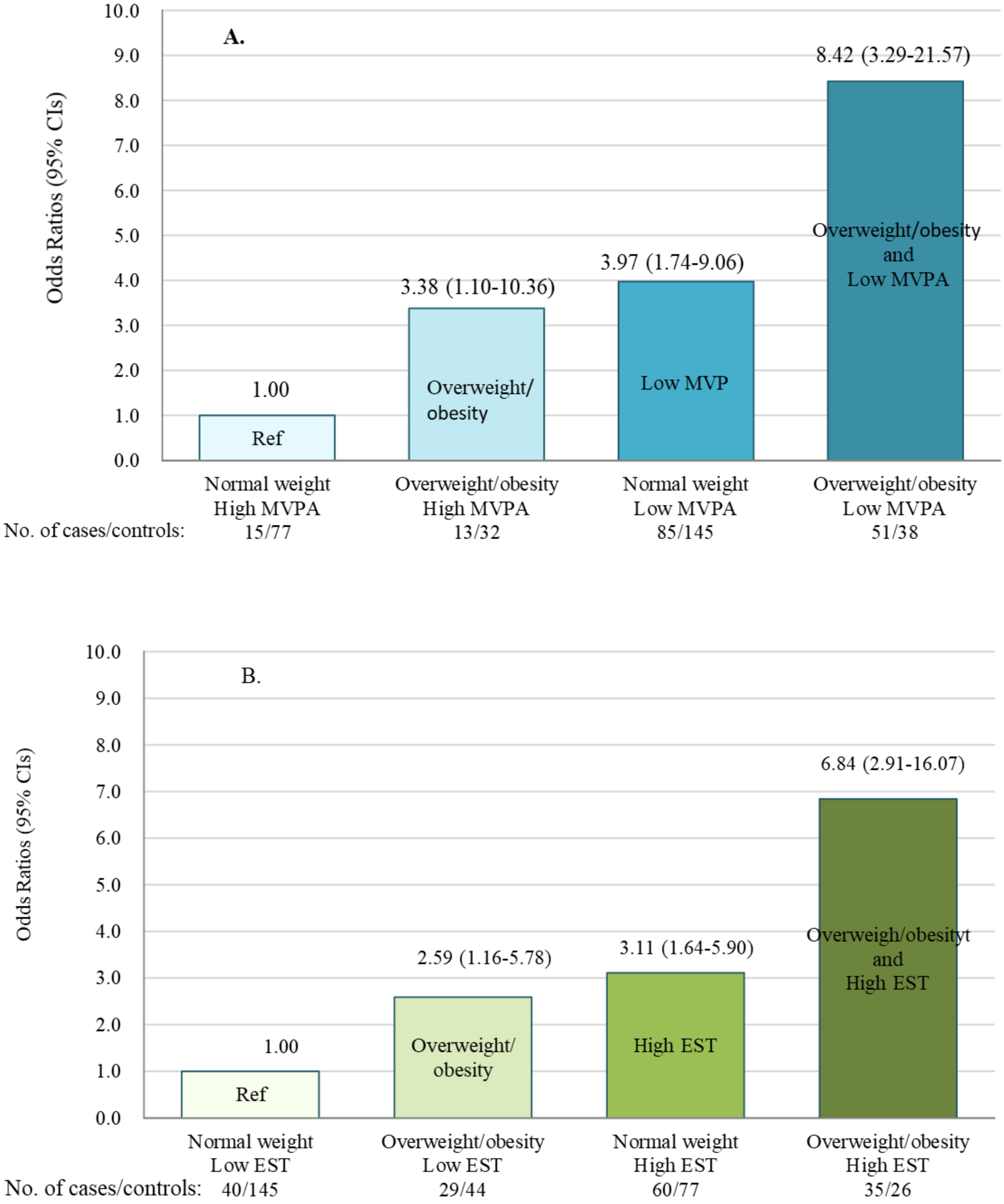

Supplement: Supplementary file 2 — Supplementary Information 2. [file 41598_2024_60345_MOESM2_ESM.pdf]
